# Chlorophyll *a* Fluorescence as a Tool in Evaluating the Effects of ABA Content and Ethylene Inhibitors on Quality of Flowering Potted *Bougainvillea*

**DOI:** 10.1100/2012/684747

**Published:** 2012-01-04

**Authors:** Antonio Ferrante, Alice Trivellini, Eva Borghesi, Paolo Vernieri

**Affiliations:** ^1^Department of Plant Production, Università degli Studi di Milano, 20133 Milano, Italy; ^2^Department of Crop Biology, University of Pisa, 56124 Pisa, Italy

## Abstract

Flowering potted plants during the postproduction stage are usually stored in inadequate environmental conditions. We evaluated the effect of the most common storage conditions and treatments on two *Bougainvillea* cultivars after harvest and during recovery. Flowering potted *Bougainvillea* plants were treated with 100 mL 2 mM amino-oxyacetic acid (AOA) or 500 ppb 1-methylcyclopropene (1-MCP) prior storage in dark at 14°C for simulating transport or storage conditions and, subsequently, transferred to growth chambers at 20°C in the light for one week for evaluating the recovery ability. The plant stress during the experiments was assessed by ethylene, ABA, and chlorophyll *a* fluorescence measurements. Ethylene production was affected by temperature rather than treatments. ABA concentration declined in leaves and flowers during storage and was not affected by treatments. Fluorescence parameters appear to be very useful for screening *Bougainvillea* cultivars resistant to prolonged storage periods.

## 1. Introduction

The ornamental quality of flowering potted plants depends on flowers number, longevity, turnover, and foliage (number and colour). The postproduction handling and care, such as storage conditions, are crucial for plants marketability at final markets [1]. *Bougainvillea* plants during storage and transportation often suffer of flower/bracts drop. Plant hormones such as abscisic acid and ethylene can determine quality reduction in different plant species. Ethylene usually induces leaf yellowing, flower senescence, wilting, and abscission in sensitive plants [2]. Different chemical compounds are able to reduce or inhibit ethylene biosynthesis but do not protect plants if ethylene is already present in the storage or transportation environments as pollutant [3]. The complete protection from ethylene can be obtained using ethylene action inhibitors such as silver thiosulfate (STS) or 1-methylcyclopropene (1-MCP). Therefore, STS has been widely used for protecting plants after harvest or during postproduction stage [4]. The 1-MCP has also been effective in reducing quality losses in many ornamental species [2, 5]. In *Bougainvillea* potted plants, auxins applied alone or in combination with STS prevented bracts drop [4]. Ethanol treatments at concentrations of 8 and 10% also extended the vase life of cut *Bouganivillea* inflorescences [6].

Abscisic acid (ABA) accumulation in flowers or leaves of ornamental plants usually negatively affects quality [7]. However, little information is available on the ABA role in post-production ornamental flowering plants.

Usually, at the onset, physiological stresses are not visible and often when symptoms appear, plant quality is almost compromised. Therefore, it is very interesting to identify non destructive measurements that allow an early detection of stress conditions, during and immediately after storage or transportation. The chlorophyll *a* fluorescence and derivate indexes are good markers of plant stress conditions, widely used across plant physiology studies. The application in post-production of ornamentals has been firstly reported for potted foliage plants such as *Ficus benjamina *L.,* Dieffenbachia picta *Schott.,* Codiaeum variegatum *L., and cut rose flowers [8].

Chlorophyll *a* fluorescence is firstly affected when plants are exposed to adverse environmental conditions, and this can be correctly extended to postharvest or post-production conditions [9]. Chlorophyll *a* fluorescence has been used for evaluating the quality of lamb's lettuce during storage at different temperatures such as 4 or 10°C. Results demonstrated that the maximum quantum efficiency of PSII (F_*v*_/F_*m*_) and the most part of JIP indexes changed with storage time and temperature [10, 11]. Chlorophyll *a* fluorescence has been also used for evaluating the effects of preservative solutions on postharvest performance of cut flowers such as *Bougainvillea* [6, 12] and stock flowers [11]. In cut foliage, the chlorophyll *a* fluorescence was used for evaluating the best storage conditions for preserving quality and maximizing the vase life [13]. Great information can be obtained from the JIP test which provides biophysical parameters derived by the analysis of intermediate data point of the fluorescence induction curve and quantifies the PSII behaviour [14–16]. The JIP test can be used to explain the stepwise flow of energy through PS II at the reaction centre or cross-section of area exposed to exciting light [17].

The aim of this work was to study the quality changes after storage or transport of two *Bougainvillea* cultivars with different storage attitudes. Ethylene inhibitors were applied before experiments started with aim to limit post-production stresses during storage or transportation. Plant hormones, relative water content, chlorophyll content, and chlorophyll *a* fluorescence were monitored for evaluating plant stress and quality. Chlorophyll *a* fluorescence was used to quantify the stress of treated and control plants. Fluorescence parameters have been evaluated as potential markers for quality estimation of potted plants before and after storage or transportation.

## 2. Materials and Methods

### 2.1. Plant Materials

Flowering potted *Bougainvillea* “Rosenka” and *B. *“Don Mario” were bought at commercial stage at a local nursery and grown for three weeks under natural greenhouse conditions before storage/transportation simulation. The experiments were performed in two years in the period of April-May (Pisa, Italy). Plants were regularly irrigated until 24 h before experiments began, in order to avoid the presence of free water during the storage/transportation simulation, since free water may induce gray mold development even at low temperature.

### 2.2. Treatments, Storage/Transport Simulation, and Recovery

Flowering potted plants were treated with ethylene inhibitors: 100 mL 2 mM amino-oxyacetic acid (AOA) or 500 ppb 1-methylcyclopropene (1-MCP). The AOA was applied as foliar spray. Control plants and 1-MCP-treated plants were sprayed with distilled water as well. The 1-MCP treatment was applied in air-tight growth chamber for 24 h. Plants were tightly wrapped in plastic film, following the standard commercial procedures, and stored in growth chamber at 14°C with 70–80% relative humidity (RH) in the dark. Subsequently, plants were transferred in growth chamber equipped with photoperiod of 12 h light/dark cycle, 100 *μ*mol m^−2^ s^−1^ light intensity, and 60–70% RH for recovery ability evaluation.

### 2.3. Relative Water Content, Total Chlorophyll, and Chlorophyll a Fluorescence Determinations

Relative water content (RWC) was determined as stated by Slatyer [18] and expressed as percentage of water content at a given time and tissue as related to the water content at full turgor.

RWC = (FW − DW)/(TW − DW), where FW is fresh weight, DW is dry weight, and TW is turgid weight. Leaf discs were collected from 3 to 4 leaves and immediately wrapped in Petri dishes to minimize evaporation. Samples were stored in dark and weighted for FW determination. Leaf pieces were immediately weighed, then immersed in deionised water for 24 h, and kept away from physiological activity by physical inhibition of growth and respiration (dark incubation in fridge). Thereafter, the TW was determined, the leaf pieces were blotted to dryness and reweighted, and then placed in a dryer at 70°C (3 days) for DW determination.

Total leaf chlorophyll was extracted using 99.9% methanol as solvent. Samples were kept in dark at 4°C for 24 h. Quantitative chlorophyll determinations were carried out immediately after extraction. Absorbance was measured spectrophotometrically (Shimadzu UV-Vis 1204, Tokio, Japan). Chlorophyll contents were calculated by Lichtenthaler's formula [19].

Chlorophyll *a* fluorescence transients were determined on dark-adapted leaves kept for 30 min at room temperature, using a portable Handy PEA (Hansatech, UK). The measurements were taken on the leaf surface (4 mm diameter) exposed to an excitation light intensity (ultrabright red LEDs with a peak at 650 nm) of 3000 *μ*mol m^−2^ s^−1^ (600 W m^−2^) emitted by three diodes. Leaf fluorescence detection was measured by fast-response PIN photodiode with RG9 long-pass filter (Hansatech, technical manual). The parameters measured were F_*o*_, F_*m*_, and F_*v*_/F_*m*_. The JIP test on the intermediate points of the fluorescence induction curves was carried out, and phenomenological and specific indexes were calculated [14].

### 2.4. Abscisic Acid and Ethylene Determination

ABA was determined by an indirect ELISA based on the use of DBPA1 monoclonal antibody and raised against S(+)-ABA [20]. The ELISA was performed according to the method described by Walker-Simmons [21], with minor modifications.

Leaf or flowers samples (100 mg FW) were collected, weighted, frozen in liquid nitrogen, and then stored at −80°C until analysis. ABA was measured after extraction in distilled water (water : tissue ratio = 10 : 1 v : w) overnight at 4°C. Plates were coated with 200 *μ*L per well ABA-4′-BSA conjugate and incubated overnight at 4°C and then washed three times with 75 mM PBS buffer, pH 7.0, containing 1 g L^−1^ BSA and 1 mL L^−1^ Tween 20, keeping the third washing solution for 30 min at 37°C. Then, 100 *μ*L ABA standard solution or sample and, subsequently, 100 *μ*L DBPA1 solution (lyophilized cell culture medium diluted in PBS buffer containing 10 g L^−1^ BSA and 0.5 mL L^−1^ Tween 20, at a final concentration of 50 *μ*g mL^−1^) were added to each well, and competition was allowed to occur at 37°C for 30 min. Plates were then washed again as described above, and 200 *μ*L per well of secondary antibody (Alkaline phosphatase-conjugated rabbit anti-mouse, Sigma, Italy) in PBS buffer containing 10 g L^−1^ BSA and 0.5 mL L^−1^ Tween 20, at a final dilution of 1 : 2000 was added and incubated for 30 min at 37°C. Plates were washed again, and 200 *μ*L per well p-Nitrophenyl phosphate was added and incubated for 30 min at 37°C. Absorbance readings at 415 nm were obtained using an MDL 680 Perkin-Elmer microplate reader. For each treatment, four independent samples were assayed in triplicate.

Ethylene production was measured by enclosing leaves or flowers in air-tight containers (250 mL). Two mL gas samples were taken from the headspace of the containers after 1 h incubation at room temperature. The ethylene concentration in the sample was measured by a gas chromatograph (HP5890, Hewlett-Packard, Menlo Park, Calif) using a flame ionization detector (FID), a stainless steel column (150 × 0.4 cm ø packed with Hysep T), column and detector temperatures of 70°C and 350°C, respectively, and nitrogen carrier gas at a flow rate of 30 mL min^−1^.

### 2.5. Statistical Analysis

Experiments were organised in randomised blocks, and five plants were used for each treatment. The data in tables and figures are means and standard errors (*n* = 5). Data were subjected to two-way ANOVA analysis. Differences among means were determined using Bonferroni's posttest.

## 3. Results

### 3.1. Flower and Leaf Losses

The flower and leaf losses were not affected by treatments in both cultivars. The weight of flowers lost was in average of 8.99 and 3.13 g FW in cv. Don Mario and cv. Rosenka, respectively (Table 1). The weight of leaves lost was in average of 6.1 and 0.97 g FW in cv. Don Mario and cv. Rosenka, respectively.

### 3.2. Chlorophyll Content and Relative Water Content

**Figure 1:**
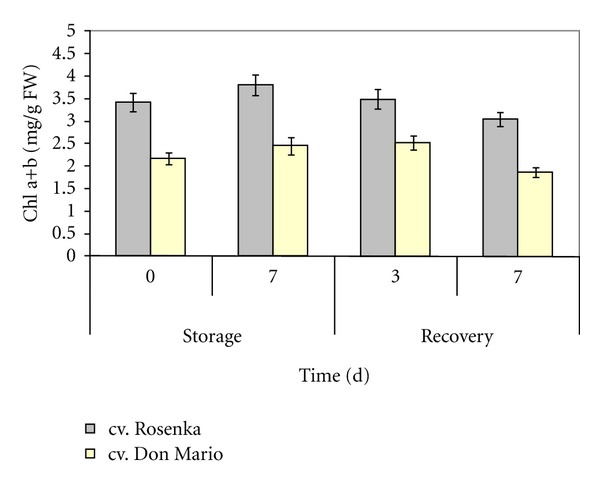
Total chlorophyll of flowering *Bougainvillea* potted plants during storage and recovery. Values are means with standard errors (*n* = 5).

The two cultivars of *Bougainvillea*, Rosenka and Don Mario, had different total chlorophyll content. At the beginning of the experiment, the chlorophyll content in the leaves was 3.4 mg g^−1^ FW in cv. Rosenka and 2.2 mg g^−1^ FW in cv. Don Mario (Figure 1). In both cultivars, a slight reduction in chlorophyll content was observed after 7 days of recovery. No difference was observed among treatments.

**Figure 2:**
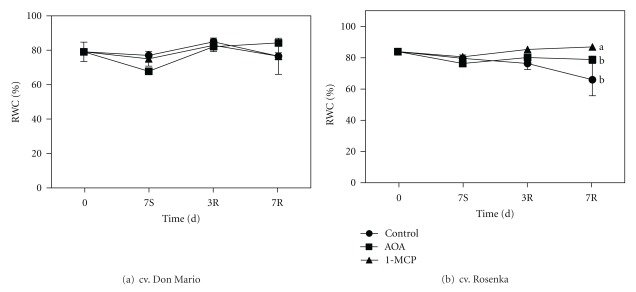
Relative water content (RWC) measured from leaves of cv. Don Mario (a) and cv. Rosenka (b) stored for 7 days (7S) at 14°C and transferred at 20°C for recovery evaluation (3R and 7R). Values are means with standard errors (*n* = 10). Data were subjected to two-way ANOVA, and different letters, when present, mean that values are statistically different, *P* < 0.05.

Relative water content (RWC) represents a useful indicator of the water balance state in a plant, essentially because it expresses the absolute amount of water, which the plant requires to reach artificial full saturation. In both cultivars, the RWC during storage did not significantly change and in average was about 80% in all treatments (Figure 2). During the recovery period and exactly after one week, the RWC was lower in control (66%) than in AOA, and 1-MCP-treated plants.

### 3.3. Ethylene and ABA Content

**Figure 3:**
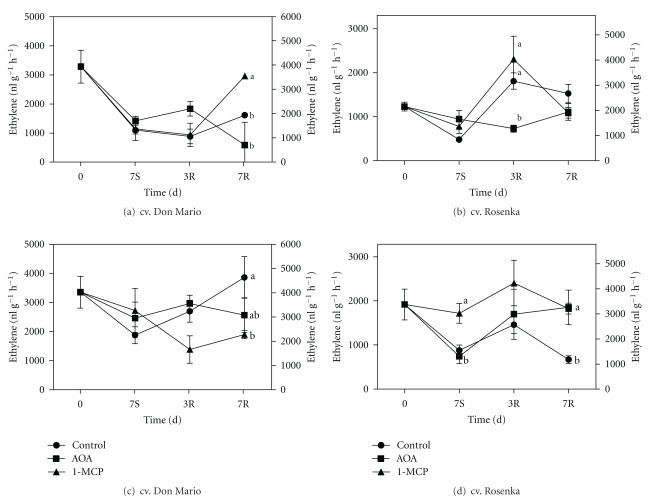
Ethylene production from leaves of cv. Don Mario (a) and cv. Rosenka (b) and from flowers of cv. Don Mario (c) and cv. Rosenka (d) stored for 7 days (7S) at 14°C and transferred at 20°C for recovery evaluation (3R and 7R). Values are means with standard errors (*n* = 10). Data were subjected to one-way ANOVA, and different letters, when present, mean that values are statistically different, *P* < 0.05.

At the beginning of the experiment, hormonal changes are compared between the two cultivars. Ethylene production from leaves was 2.6-fold higher in cv. Don Mario compared to cv. Rosenka. After seven days of storage, the ethylene production in cv. Don Mario leaves declined by 57–67% among treatments (Figure 3(a)). Treatments with ethylene inhibitors did not affect the ethylene production during storage but influenced the ethylene biosynthesis during the recovery period, especially after seven days (Figure 3(a)). After storage, the leaves of 1-MCP-treated plants showed an increase of ethylene production, reaching the initial values. The AOA-treated leaves, instead, after seven days showed the lowest values.

The leaves of cv. Rosenka plants treated with AOA showed the same ethylene production either during or after storage simulation. On the contrary, 1-MCP-treated plants showed the same behaviour of control ones, with an increase of ethylene biosynthesis after 3 days of recovery followed by a decrease until reaching the initial values after seven days (Figure 3(b)).

The ethylene production of flowers taken from control plants of cv. Don Mario declined during storage and linearly increased during the recovery period. In treated plants, instead, the ethylene production of flowers declined significantly in 1-MCP treatment until 3 days of recovery then remained unchanged, while flowers harvested from AOA-treated plants did not show significant variation during storage or recovery period (Figure 3(c)).

In the cv. Rosenka, flowers sampled from control and AOA treatment showed a strong reduction in ethylene biosynthesis during storage. After storage, the ethylene production increased again, and after three days in growth chamber, the production of the hormone was similar in control and AOA treatment. After one week of recovery, the ethylene dropped in the control, while in AOA it remained unchanged. The ethylene production from flowers harvested from 1-MCP treatment did not show any significant change during the whole experimental period (Figure 3(d)).

**Figure 4:**
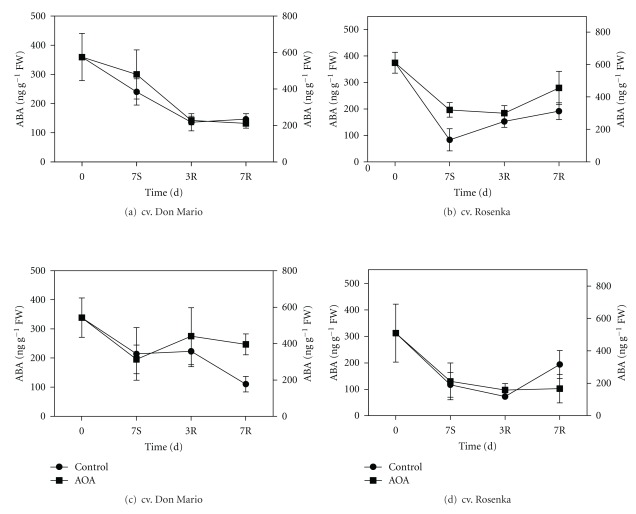
Endogenous ABA content from leaves of cv. Don Mario (a) and cv. Rosenka (b) and from flowers of cv. Don Mario (c) and cv. Rosenka (d) stored for 7 days (7S) at 14°C and transferred at 20°C for recovery evaluation (3R and 7R). Values are means with standard errors (*n* = 10). Data were subjected to one-way ANOVA, and different letters, when present, mean that values are statistically different, *P* < 0.05.

The endogenous ABA content declined during storage in all treatments, in both organs and cultivars. In leaves, the ABA content in both cultivars at beginning of experiments ranged from 369 to 375 ng g^−1^ FW (Figures 4(a) and 4(b)). After 7 days of storage in cv. Don Mario no differences were found between control and treatments (Figure 4(a)). After three days of recovery, the ABA content in cv. Don Mario leaves was even lower with values similar among treatments and remained constant after one week. In the cv. Rosenka, the ABA concentration in leaves also declined in all treatments during storage. Leaves of controls had lower ABA content compared to those of AOA and 1-MCP treatments (Figure 4(b)). During the recovery period, an increase of ABA content was observed in controls and AOA treatment, while in 1-MCP, the level of ABA remained constant.

Between the different plant tissues, flowers had the highest amount of ABA that was 845 ng g^−1^ FW in cv. Don Mario and 510 ng g^−1^ FW in cv. Rosenka at the beginning of experiments (Figures 4(c) and 4(d)).

The ABA concentration in open flowers (bracts and true flower) of cv. Don Mario dropped in all treatments after seven days of storage. The lowest value was found in flowers harvested from 1-MCP-treated plants. During the recovery period, a slight increase of ABA was observed in AOA and 1-MCP treatments. In open flowers of cv. Rosenka, the ABA concentration declined during storage only in controls and AOA, while in flowers harvested from 1-MCP-treated plants, the reduction was also observed during the recovery period. After one week in growth chamber, the ABA level increased again in control flowers (Figure 4(d)).

### 3.4. Chlorophyll a Fluorescence Parameters

Chlorophyll fluorescence measurements were carried out for evaluating the effects of treatments on plant behaviour during and after storage. The fluorescence level, when plastoquinone electron acceptor pool (Qa) is fully oxidised, F_*o*_ measured at *F*_20*μs*_ was 715 in cv. Rosenka and 648 in cv. Don Mario. After storage and recovery no significant differences were observed in both cultivars (see Supplementary Data in Supplementary material available online at doi:10.1100.2012.684747). The F_*m*_ values in the cv. Don Mario were significantly lower in leaves of AOA treated plants, while no difference was observed between control and 1-MCP treatment. After seven days of storage the F_*m*_ declined in all treatments and increased again during recovery, overcoming the initial values (Table 2).

The F_*m*_ in cv. Rosenka did not change during storage, while increased in all treatments during recovery period, from 11 to 17%. The highest F_*m*_ values were recorded in control plants (Table 2).

**Figure 5:**
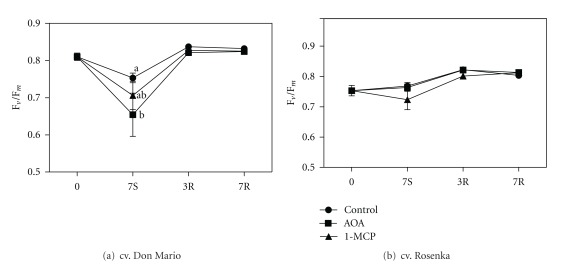
Maximum quantum efficiency of photosystem II (F_*v*_/F_*m*_) from leaves of cv. Don Mario (a) and cv. Rosenka (b) stored for 7 days (7S) at 14°C and transferred at 20°C for recovery evaluation (3R and 7R). Values are means with standard errors (*n* = 10). Data were subjected to one-way ANOVA, and different letters, when present, mean that values are statistically different, *P* < 0.05.

The F_*v*_/F_*m*_ ratio in cv. Don Mario leaves declined during storage in all treatments. It was statistically lower in AOA-treated leaves. After seven days of storage, the F_*v*_/F_*m*_ values were 0.75, 0.65, and 0.70 in control, AOA, and 1-MCP treatments, respectively. During the recovery period, the F_*v*_/F_*m*_ ratio increased in all treatments and ranged from 0.82 to 0.83 (Figure 5(a)).

In the cv. Rosenka, the F_*v*_/F_*m*_ ratio did not significantly change during storage, even if a slight decline was observed in 1-MCP-treated plants. As found in cv. Don Mario, also in cv. Rosenka the F_*v*_/F_*m*_ ratio increased with values higher than those recorded at the beginning of the experiments (Figure 5(b)).

**Figure 6:**
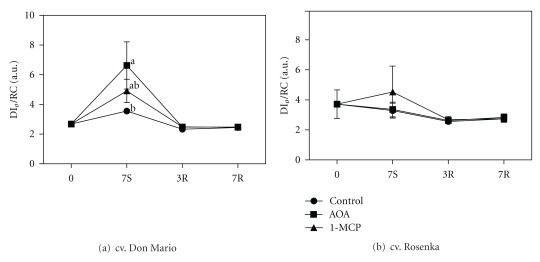
Dissipation energy per active reaction center (DI_*o*_/RC) measured from leaves of cv. Don Mario (a) and cv. Rosenka (b) stored for 7 days (7S) at 14°C and transferred at 20°C for recovery evaluation (3R and 7R). Values are means with standard errors (*n* = 10). Data were subjected to one-way ANOVA and different letter, when present, mean that values are statistically different *P* < 0.05.

The fluorescence data collected from the two cultivars in the three treatments were used to perform the JIP test. The most informative indexes that showed significant differences among treatments, during storage or recovery, are reported in the text; the others can be found in the Supplementary Data. The JIP test was performed since it provides information on energy flux and potential photosynthesis activity. The energy dissipated per reaction center (DI_*o*_/RC) in the cv. Don Mario plants increased during storage, and the highest value was observed in the AOA treatment, while in 1-MCP the increase was not significant. During the recovery period, the DI_*o*_/RC index declined, and values were similar to those found at the beginning of the experiment (Figure 6(a)). In the cv. Rosenka, the DI_*o*_/RC was higher at the beginning of the experiment than in cv. Don Mario, but did not change among treatments during storage and recovery (Figure 6(b)). The dissipation energy expressed per cross-section (DI_*o*_/CS) was not influenced by treatments at any time during the experiments (Supplementary Data).

**Figure 7:**
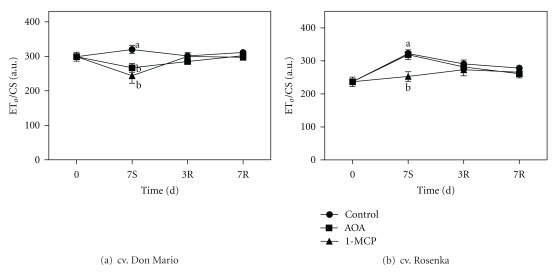
Electron transport flux per CS (ET_o_/CS) measured from leaves of cv. Don Mario (a) and cv. Rosenka (b) stored for 7 days (7S) at 14°C and transferred at 20°C for recovery evaluation (3R and 7R). Values are means with standard errors (*n* = 10). Data were subjected to one-way ANOVA and different letters, when present, mean that values are statistically different *P* < 0.05.

The electron flux per cross-section (ET_o_/CS) declined in the treatments with AOA and 1-MCP during storage in cv. Don Mario (Figure 7(a)). However, the ET_o_/CS during recovery increased, reaching the initial values in both ethylene inhibitor treatments.

In the cv. Rosenka, the ET_o_/CS increased during storage, especially in control and AOA treatments (Figure 5(b)). After the recovery period, no differences were found among treatments.

The electron flux expressed per active reaction center (ET_o_/RC), instead, did not statistically change in both cultivars and treatments (Supplementary Data).

**Figure 8:**
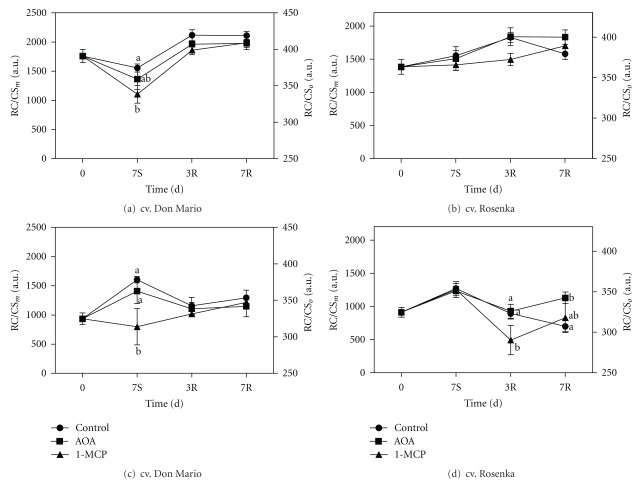
Density of reaction centers per CS at F_*m*_ (RC/CS_*m*_) measured from leaves of cv. Don Mario (a) and cv. Rosenka (b) and density of reaction centers per CS at F_*o*_ (RC/CS_o_) measured from leaves of cv. Don Mario (c) and cv. Rosenka (d) stored for 7 days (7S) at 14°C and transferred at 20°C for recovery evaluation (3R and 7R). Values are means with standard errors (*n* = 10). Data were subjected to one-way ANOVA, and different letterss, when present, mean that values are statistically different, *P* < 0.05.

The density of reaction centers per cross-section at F_*m*_ (RC/CS_*m*_) in the cv. Don Mario decreased in all treatments in both cultivars. The strongest reduction was observed in the 1-MCP treatment (Figure 8(a)). During recovery, F_*m*_ (RC/CS_*m*_) increased in all treatments, showing values higher than those found at the beginning of the experiment. The cv. Rosenka had lower active reaction centers (1450) compared to cv. Don Mario (1800) and did not show any significant variation in RC/CS_*m*_ during storage, and only a slight increase was observed during recovery (Figure 8(b)). The density of reaction centers per cross-section at F_20*μs*_ in cv. Don Mario increased in control- and AOA-treated plants, while 1-MCP-treated ones, it did not show any significant change compared to the initial values (Figure 8(c)). cv. Rosenka plants treated with 1-MCP had lower values of RC/CS_*o*_ after 3 days of recovery, then values for this parameter increased reaching the same level of control plants (Figure 8(d)). However, in both cultivars, the RC/CS_*o*_ values were fully restored after 7 days of recovery.

**Figure 9:**
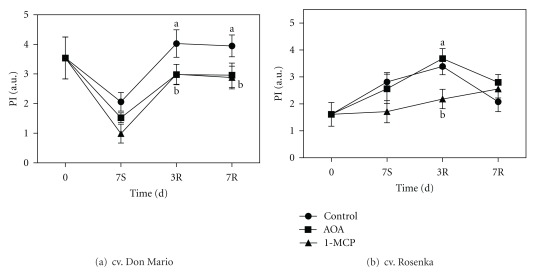
Performance index (PI) measured from leaves of cv. Don Mario (a) and cv. Rosenka (b) stored for 7 days (7S) at 14°C and transferred at 20°C for recovery evaluation (3R and 7R). Values are means with standard errors (*n* = 10). Data were subjected to one-way ANOVA, and different letters, when present, mean that values are statistically different, *P* < 0.05.

The performance index (PI) in the cv. Don Mario declined during storage (Figure 9(a)). PI values were not different among treatments, but 1-MCP treatment showed the lowest values. During the recovery period, the PI increased and plants treated with AOA and 1-MCP had lower values compared with control.

In the Rosenka cultivar, the PI increased during storage and the first three days of recovery in AOA and control plants, reaching values of 3.7-3.8 (Figure 9(b)). The PI in 1-MCP treatment was not affected by the storage period and increased during the recovery period reaching the same values of control and AOA treatment.

## 4. Discussion

The quality of flowering potted plants depends on the visual appearance. It mainly derives from number of open flowers and leaf health status. On the basis of results obtained, the cv. Don Mario lost higher amount of flowers/bracts and leaves compared to cv. Rosenka, confirming its low aptitude to storage and transportation. The chlorophyll content is responsible for the greenness, and it represents an important quality parameter. In the two cultivars, chlorophyll content was not affected by storage; therefore, it does not represent a post-production problem for these two *Bougainvilleas*.

The RWC expresses the percentage of water content at a given time and tissue related to the water content at full turgor. Results indicated that only 1-MCP had a positive effect on the water content after one week of recovery. As Kawakami et al. [22] recently reported, water-stressed cotton plants treated with 1-MCP had a higher stomatal resistance, less negative water potential, and higher and better maintenance of membrane integrity.

Ethylene is a plant hormone always associated with stresses and is used as quality marker in postharvest field. Unfortunately, during postproduction of potted plants, the environmental conditions along the distribution chain may change continuously, and ethylene production is strongly affected by environment temperature. Ethylene induces flowers shattering, petal discoloration, and leaf yellowing on several potted plants [23, 24].

In our experiments, ethylene production declined in both cultivars during the first seven days of storage. This result is probably due to the low temperature (14°C) used during storage, compared to the growing temperatures of 25–28°C in the greenhouse. In fact, it is well known that ethylene evolution is temperature-dependent and linearly increases from 15°C to 25°C.

The increase of ethylene production during the recovery period in flowers of both cultivars is probably due to an increase of 1-aminocyclopropane-1-carboxylic acid (ACC) that is not converted in ethylene by the ACC synthase and oxidase enzymes which are temperature dependent. After storage or when plants are transferred to higher temperature, the ACC is rapidly converted to ethylene [25–27].

*Bougainvillea* is a sensitive species to exogenous ethylene; in fact, treatments with STS prevented bracts abscission [4, 28]. Ethanol, another ethylene biosynthesis inhibitor, was also used for preventing bracts abscission [6, 29, 30]. In our experiments, 1-MCP was tested as potential treatment for improving the quality of *Bougainvillea* after storage, but it did not give a practical benefit, at least in long storage period such as seven days in dark.

Beside ethylene, another hormone involved in flower senescence is ABA that in many plant species is associated with senescence and ornamental quality losses [31, 32]. In *Bougainvillea* potted plants, endogenous ABA declined during the storage, but its concentration did not promptly increase during the recovery period. However, no correlations were observed with the overall quality of plants.

Chlorophyll *a* fluorescence instead is a good marker for evaluating the stress conditions of flowering potted plants since this parameter is able to show the most part of stress. Chlorophyll *a *fluorescence and derivate JIP test indexes are nondestructive parameters that can be used for evaluating the health status of plants evidencing a status of stress. Modulated chlorophyll *a* fluorescence has been already used for evaluating the effect of different environments on plant stress and quality [8]. In our study, continuous excitation fluorescence measurements from dark-adapted leaves were performed. Analogous postharvest studies have been performed on *Bougainvillea* flowers, where chlorophyll *a* fluorescence was measured from bracts for evaluating the effect of ethanol treatments on vase life [6, 30].

In our experiments, all parameters or derived indexes from fluorescence data collection were used for evaluating the stress of *Bougainvillea* plants after simulated storage/transport in dark conditions for one week. The JIP test was carried out on fluorescence data. In postproduction of ornamentals, the JIP parameters provide information on the energy flux status and the potential recovery of plants after storage or transportation.

The minimum fluorescence level (Fo) when a pool of plastoquinones are fully oxidised (the PSII releases all electrons), usually increases during leaf senescence, as previously demonstrated in *Cucumis sativus* cotyledons or mature stock leaves [11, 33]. Also in *Bougainvillea,* cut flowers during senescence F_*o*_ measured from petals showed an increase [6]. The fluorescence level when Qa is transiently fully reduced represents the maximum fluorescence (  F_*m*_) and also declines during leaf senescence [11]. During storage, the reduction of F_*m*_ and the maximum quantum efficiency of photosystem II (F_*v*_/F_*m*_) are commonly found, indicating the increase of stress and probably senescence [33]. The reduction F_*v*_/F_*m*_ during storage or senescence was observed for a wide range of horticultural crops such as young stored leaves [10], in fully developed leaves of cut *Eucalyptus parvifolia* foliage [13], in sweet basil stored at 4 or 8°C [34] and in cut rose flowers for evaluating low-temperature injury [35]. Usually, the F_*v*_/F_*m*_ ratio during senescence declines later since the efficiency of PSII is preserved as long as possible, while the JIP test-derived indexes are able to show destabilization of the photochemical machinery very early before the senescence processes take place [10, 36].

Finally, an overall index, PI, combines the density of reaction centres (expressed on an absorption basis), the quantum yield of primary photochemistry and the ability to provide electrons into the electron flux between photosystem II and photosystem I. These parameters represent three functional steps that regulate the initial stage of photosynthetic activity of a reaction centre complex [14, 37, 38].

In our experiments, the PI index evidenced the differences between cv. Don Mario and cv. Rosenka plants; the former resulted more sensitive to postproduction quality losses, while the latter appeared more resistant and did not seem to require any treatment for preserving leaf quality.

In this work, only the indexes that showed higher changes were taken in consideration in order to identify possible markers for quality evaluation in ornamental potted plants. The F_*v*_/F_*m*_, DI_*o*_/RC, ET_*o*_/CS, RC/CS_*m*_, and PI indexes clearly demonstrated that ethylene inhibitors increased plant stress during storage or recovery. These results suggest that in *Bougainvillea* potted plants, ethylene inhibitors do not improve plant tolerance to post-production stress conditions. AOA increased plant stress especially in cv. Don Mario even if the stress was temporary and plants recovered in three or seven days. Thus, treatments increased the dissipation energy and decreased the electron flux indicating that these ethylene inhibitors increased the stress and/or senescence. During senescence, the cell membranes lose their integrity and may affect the energy transfer. Subsequently, the electron fluxes may be interrupted with alteration of all chlorophyll *a* fluorescence parameters. However, both cultivars showed a high ability to restore the photochemical efficiency since almost parameters returned to optimal values.

In conclusion, chlorophyll *a* fluorescence data showed that cv. Rosenka plants were more resistant than cv. Don Mario, and fluorescence parameters might be used for screening *Bougainvillea* cultivars more suitable for long-distance markets that require prolonged storage periods. Ethylene inhibitors in our experimental conditions did not provide any benefit to preserving ornamental quality of *Bougainvillea* flowering potted plants. On the contrary, the fluorescence data indicated that these treatments had negative effect and enhanced the plant stress.

## Supplementary Material

Supporting material : The JIP analysis and derived indices calculated on the intermediate steps of chlorophyll *a* fluorescence measured on *Bougainvillea* cultivars treated with AOA or 1-MCP and stored at 14°C for one week in dark. The recovery ability was evaluated by keeping the pants in growth chamber for one week at 20°C. Mean values are accompanied by standard errors (*n*=5). Data were subjected to two-way ANOVA and different letters mean that values are statistically different *P*<0.05.

## Figures and Tables

**Table 1:** Flower and leaves lost after seven days storage from two *Bougainvillea* cultivars. Values are means with standard errors (*n* = 5).

	Flowers		Leaves	
Cv Don Mario	FW (g)	DW (g)	FW (g)	DW (g)
Control	8.19 ± 4.269	2.19 ± 1.099	3.96 ± 1.764	1.08 ± 0.501
AOA	10.04 ± 2.102	2.75 ± 0.468	4.97 ± 0.812	1.37 ± 0.321
1-MCP	8.70 ± 3.666	2.32 ± 1.161	9.69 ± 5.234	2.27 ± 1.267

Cv Rosenka				
Control	2.29 ± 0.800	0.60 ± 0.244	1.62 ± 0.649	0.50 ± 0.226
AOA	5.35 ± 1.575	1.90 ± 0.552	0.87 ± 0.152	0.25 ± 0.061
1-MCP	1.75 ± 1.037	0.64 ± 0.297	0.425 ± 0.425	0.15 ± 0.149

Data were subjected to one-way ANOVA analysis, and no difference was found for *P* < 0.05.

**Table 2:** F_*m*_ measured from leaves of two *Bougainvillea* cultivars stored for 7 days (7S) at 14°C and transferred at 20°C for recovery evaluation after 3 and 7 days (3R and 7R).

Time (d)	F_*m*_		
	cv. Don Mario		
	Control	AOA	1-MCP
0	3445.2 ± 73.51	3445.0 ± 73.51	3445.2 ± 73.00
7 S	3180.7 ± 112.64^a^	2520.0 ± 370.72^b^	2748.1 ± 231.00^ab^
3 R	3913.3 ± 48.04	3724.0 ± 45.63	3772.1 ± 100.00
7 R	3830.2 ± 91.45	3902.0 ± 46.61	3928.5 ± 59.00

		Cv. Rosenka	
0	2952.7 ± 102.71	2952.7 ± 102.71	2952.7 ± 102.71
7 S	3007.7 ± 83.69	2995.1 ± 101.40	2811.9 ± 243.84
3 R	3462.1 ± 50.94	3309.5 ± 76.32	3305.5 ± 87.11
7 R	3556.2 ± 53.45	3299.0 ± 90.55	3319.1 ± 57.77

Values are means with standard errors (*n* = 10). Data were subjected to one-way ANOVA, and different letter within row indicates values statistically different, (*P* < 0.05).

## References

[1] Nell TA, Barrett JE (1990). Post-production handling of bedding and potted plants. *Acta Horticulturae*.

[2] Serek M, Sisler EC, Reid MS (1994). Novel gaseous ethylene binding inhibitor prevents ethylene effects in potted flowering plants. *Journal of the American Society for Horticultural Science*.

[3] Mensuali-Sodi A, Ferrante A, Tognoni F, Serra G (2005). Inhibitors of ethylene action and biosynthesis on cut carnation. *Agricoltura Mediterranea*.

[4] Cameron AC, Reid MS (1983). Use of silver thiosulfate to prevent flower abscission from potted plants. *Scientia Horticulturae*.

[5] Raffeiner B, Serek M, Winkelmann T (2009). 1-Methylcyclopropene inhibits ethylene effects in cut inflorescences and potted plants of Oncidium and Odontoglossum orchid species. *European Journal of Horticultural Science*.

[6] Hossain ABMS, Boyce AN, Osman N (2007). Postharvest quality, vase life and photosynthetic yield (chlorophyll fluorescence) of Bougainvillea flower by applying ethanol. *Australian Journal of Basic and Applied Sciences*.

[7] Ferrante A, Vernieri P, Serra G, Tognoni F (2004). Changes in abscisic acid during leaf yellowing of cut stock flowers. *Plant Growth Regulation*.

[8] van Kooten O, Mensink M, Otma E, van Doorn W (1991). Determination of the physiological state of potted plants and cut flowers by modulated chlorophyll fluorescence. *Acta Horticulturae*.

[9] DeEll JR, van Kooten O, Prange RK, Murr DP (1999). Applications of chlorophyll fluorescence techniques in postharvest physiology. *Horticulture Review*.

[10] Ferrante A, Maggiore T (2007). Chlorophyll a fluorescence measurements to evaluate storage time and temperature of Valeriana leafy vegetables. *Postharvest Biology and Technology*.

[11] Ferrante A, Mensuali-Sodi A, Serra G (2009). Effect of thidiazuron and gibberellic acid on leaf yellowing of cut stock flowers. *Central European Journal of Biology*.

[12] Balas J, Coronado PAG, Silva JAT, Jayatilleke MP, da Silva JT (2006). Supporting post-harvest performance of cut-flowers using fresh-flower-refreshments and other vase-water-additives. *Floriculture, Ornamental and Plant Biotechnology: Advances and Topical Issues*.

[13] Pacifici S, Mensuali-Sodi A, Serra G, Ferrante A (2008). Comparison between conventional and vacuum storage system in cut foliage. *Acta Horticulturae*.

[14] Strasser A, Srivastava A, Tsimilli-Michael M, Yunus M, Pathre U, Mohanty P (2000). The fluorescence transient as a tool to characterize and screen photosynthetic samples. *Probing Photosynthesis: Mechanisms, Regulation and Adaptation*.

[15] Strasser A, Tsimilli-Michael M, Srivastava A, Papageorgiou GC, Govindjee (2004). Analysis of the fluorescence transient. *Chlorophyll Fluorescence: A Signature of Photosynthesis*.

[16] Strasser RJ, Srivastava A, Govindjee (1995). Polyphasic chlorophyll alpha fluorescence transient in plants and cyanobacteria. *Photochemistry and Photobiology*.

[17] Force L, Critchley C, van Rensen JJS (2003). New fluorescence parameters for monitoring photosynthesis in plants 1. The effect of illumination on the fluorescence parameters of the JIP-test. *Photosynthesis Research*.

[18] Slatyer RO (1967). *Plant-Water Relationships*.

[19] Lichtenthaler HK (1987). Chlorophylls and carotenoids: pigments of photosynthetic biomembranes. *Methods in Enzymology*.

[20] Vernieri P, Perata P, Bugnoli M (1989). Solid phase radioimmunoassay for the quantitation of abscisic acid in plant crude extracts using a new monoclonal antibody. *Journal of Plant Physiology*.

[21] Walker-Simmons M (1987). ABA levels and sensitivity in developing wheat embryos of sprouting resistant and susceptible cultivars. *Plant Physiology*.

[22] Kawakami EM, Oosterhuis DM, Snider JL (2010). Physiological effects of 1-methylcyclopropene on well-watered and water-stressed cotton plants. *Journal of Plant Growth Regulation*.

[23] Woltering EJ (1987). Effects of ethylene on ornamental pot plants: a classification. *Scientia Horticulturae*.

[24] van Doorn WG, Woltering EJ (1991). Developments in the use of growth regulators for the maintenance of post-harvest quality in cut flowers and potted plants. *Acta Horticulturae*.

[25] Barrowclough PM, Field R, Fair DJ (1991). Cold storage enhances ethylene production and reduces vase life of carnation flower. *New Zealand Journal of Crop and Horticultural Science*.

[26] Serrano M, Martinez-Madrid MC, Riquelme F, Romojaro F (1995). Enhanced ethylene synthesis in cold stored carnation flowers. *Acta Horticulturae*.

[27] Vilaplana R, Soria Y, Valentines MC, Larrigaudière C (2007). Specific response of apple skin and pulp tissues to cold stress and 1-MCP treatment. *Postharvest Biology and Technology*.

[28] Gago CML, Montiero JA (2011). NAA and STS effects on bract survival time, carbohydrate content, respiration rate and carbohydrate balance of potted Bougainvillea spectabilis willd. *Postharvest Biology and Technology*.

[29] Mencarelli F, Hugo L (1991). Control of flower and bract abscission of Bougainvillea branches by ethanol solutions. *Agricoltura Mediterranea*.

[30] Hossain ABMS, Boyce AN, Majid HMA (2008). Vase life extension and chlorophyll fluorescence yield of Bougainvillea flower as influenced by ethanol to attain maximum environmental beautification as ornamental components. *American Journal of Environmental Sciences*.

[31] Ferrante A, Vernieri P, da Silva JT (2006). Abscisic acid and cut flower senescence. *Floriculture, Ornamental and Plant Biotechnology: Advances and Topical Issues*.

[32] Trivellini A, Ferrante A, Vernieri P, Mensuali-Sodi A, Serra G (2011). Effects of promoters and Inhibitors of ethylene and ABA on flower senescence of Hibiscus rosa-sinensis L. *Journal of Plant Growth Regulation*.

[33] Prakash JSS, Srivastava A, Strasser RJ, Mohanty P (2003). Senescence-induced alterations in the photosystem II functions of Cucumis sativus cotyledons: probing of senescence driven alterations of photosystem II by chlorophyll a fluorescence induction O-J-I-P transients. *Indian Journal of Biochemistry and Biophysics*.

[34] Meir S, Salim S, Chernov Z, Philosoph-Hadas S (2007). Quality improvement of cut flowers and potted plants with postharvest treatments based on various cytokinins and auxins. *Acta Horticulturae*.

[35] Pompodakis NE, Terry LA, Joyce DC, Lydakis DE, Papadimitriou MD (2005). Effect of seasonal variation and storage temperature on leaf chlorophyll fluorescence and vase life of cut roses. *Postharvest Biology and Technology*.

[36] Baldassarre V, Cabassi G, Ferrante A (2011). Use of chlorophyll a fluorescence for evaluating the quality of leafy vegetables. *Australian Journal of Crop Science*.

[37] Thach LB, Shapcott A, Schmidt S, Critchley C (2007). The OJIP fast fluorescence rise characterizes Graptophyllum species and their stress responses. *Photosynthesis Research*.

[38] Cascio C, Schaub M, Novak K, Desotgiu R, Bussotti F, Strasser RJ (2010). Foliar responses to ozone of Fagus sylvatica L. seedlings grown in shaded and in full sunlight conditions. *Environmental and Experimental Botany*.

